# Hanging Middle Turbinates: An Uncommon Complication Following Septoplasty

**DOI:** 10.7759/cureus.102903

**Published:** 2026-02-03

**Authors:** Alreem A Al-Qahtani, Emad Al Duhirat, Ahmed Shaikh

**Affiliations:** 1 Otolaryngology - Head and Neck Surgery, Hamad General Hospital, Doha, QAT; 2 School of Medicine, Qatar University, Doha, QAT; 3 Otolaryngology - Head and Neck Surgery, Hamad Medical Corporation, Doha, QAT

**Keywords:** complications, computed tomography (ct), endoscopic septoplasty, middle turbinate anatomy, revision septoplasty

## Abstract

Septoplasty is a commonly performed procedure with known complications including infection, bleeding, ocular injury, septal abscess, septal perforation, and long-term sequelae; however, middle turbinate involvement is rare and sparsely described in the literature. We report the case of a 20-year-old woman with no significant medical history who underwent septoplasty with submucosal diathermy of the inferior turbinates at a private facility and subsequently presented with persistent nasal obstruction. Computed tomography demonstrated bilaterally hanging middle turbinates. The patient was managed with revision septoplasty, bilateral excision of the hanging middle turbinates with hemostasis, and repeat submucosal diathermy of the inferior turbinates, resulting in an uncomplicated postoperative course and complete symptomatic improvement. This case underscores the importance of preoperative computed tomography in revision septoplasty to delineate nasal anatomy and guide appropriate surgical management.

## Introduction

Septoplasty is a well-known procedure that is done for patients complaining of nasal blockage due to a deviated nasal septum. The surgery is mainly done through the nasal cartilage without manipulating other structures. One of the nearby anatomies is the middle turbinate. It is a mucosa-lined bony structure of ethmoidal origin along the lateral nasal wall. It helps direct nasal airflow and condition inspired air, and its close relationship to the osteomeatal region makes its position important for normal sinus ventilation and drainage. Alteration of its normal anatomy can impair both nasal airflow and sinus function. An estimated 20% of the general population is affected by a deviated nasal septum, and among them, 25% report experiencing challenges with breathing [1]. Even though septoplasty is a simple procedure, some complications can happen in the perioperative phase like infection (3%) and bleeding (6%) [1], pain and discomfort, ocular complications, septal abscess rates from 0.4% to 12.0%, and nasal septal perforation ranging from 1.6% to 6.7% [2]. Long-term complications, such as persistence of nasal airway obstruction requiring revision surgery, saddle nose deformity, anosmia, empty nose syndrome, cerebrospinal fluid (CSF) leakage, and even blindness and rarely oronasal fistula, are not well-studied in the literature [3,4]. In terms of these reported complications, there is no case report or literature that has discussed post-operative middle turbinates hanging in the nasopharynx as a complication of post-septoplasty operation. 

## Case presentation

**Figure 1:**
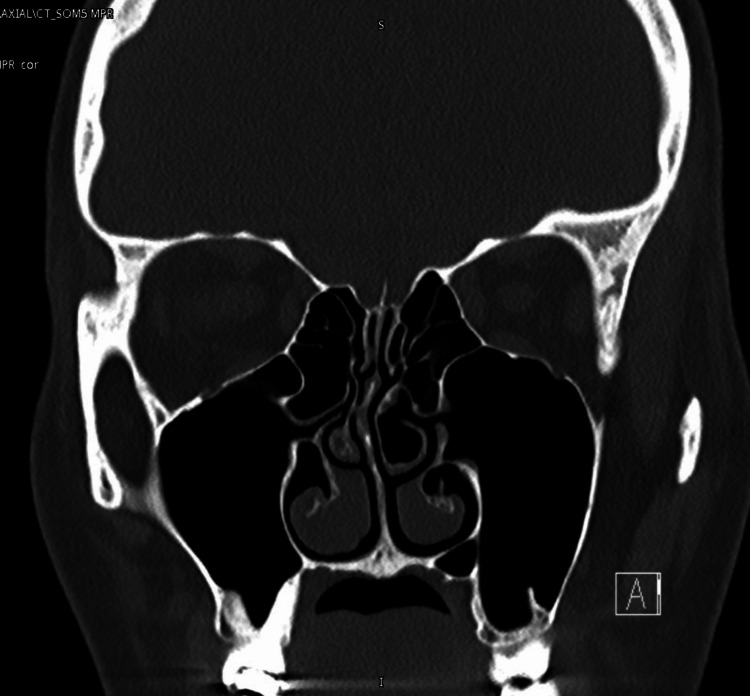
Preoperative CT scan (coronal view)

A woman in her 20s with no past medical history presented to the otolaryngology clinic with a complaint of nasal blockage. She had been coming to the ENT clinic since she was 15 years old with nasal blockage and a deviated nasal septum, as shown in preoperative CT (Figure 1).

She was advised to wait until she had completed full nasal growth. While waiting for her surgery, she decided to seek a private opinion when she was over 18, and she underwent septoplasty with submucosal diathermy of inferior turbinates with concha bullosa excision in a private facility.

**Figure 2:**
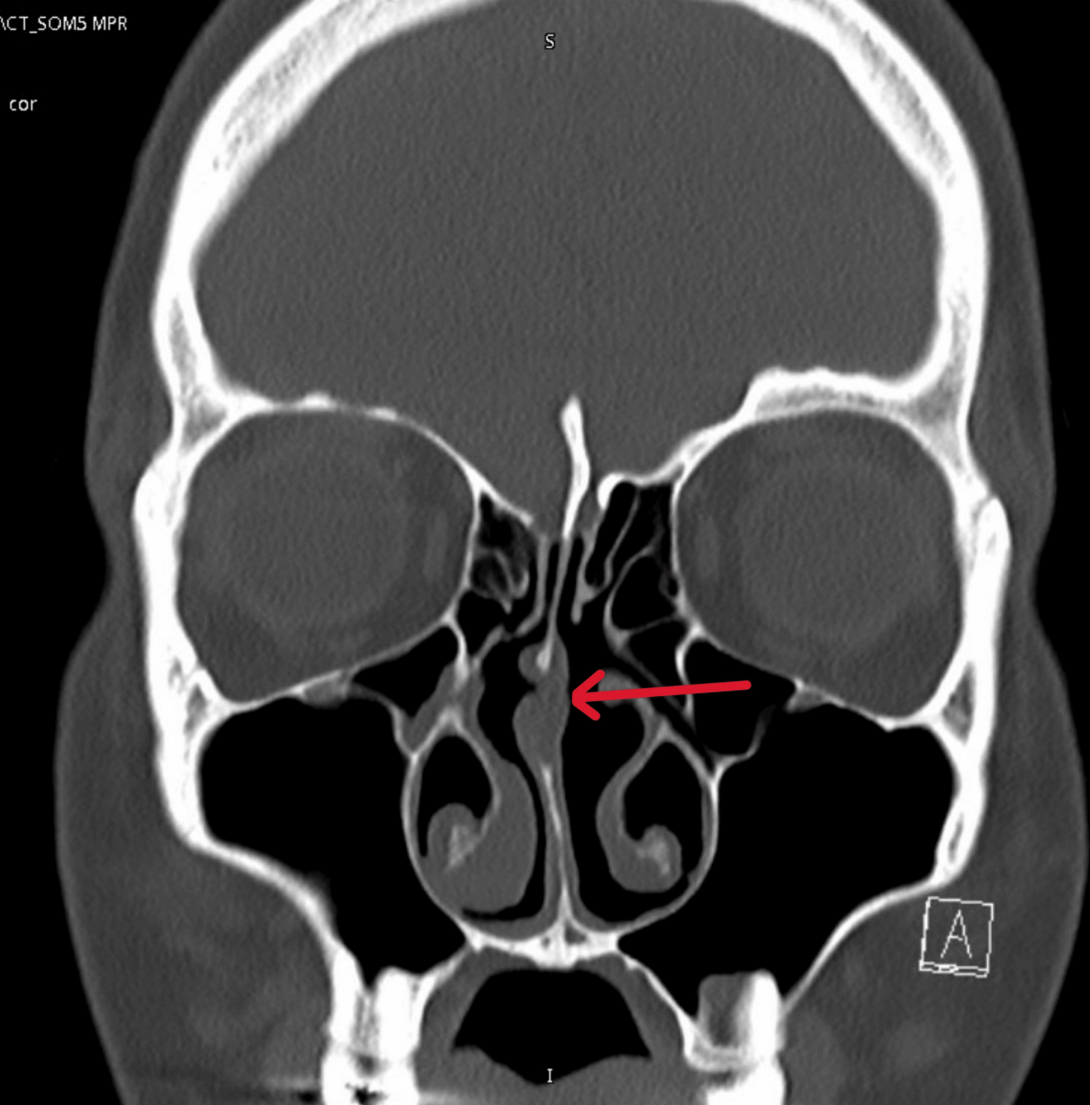
Postoperative CT scan. Note the missing cartilage (red arrow)

After her surgery, she came again, still complaining of complete nasal blockage and upon examination at that time, there was still a mild deviated nasal septum to the right, with tip drop, severe allergic mucosa, and no polyps seen, with collapsing middle turbinates in the post-nasal space. Investigation showed high IgE with 135 with positive Phadiatop. Another CT done in our facility confirmed the examination (Figure 2).

**Figure 3:**
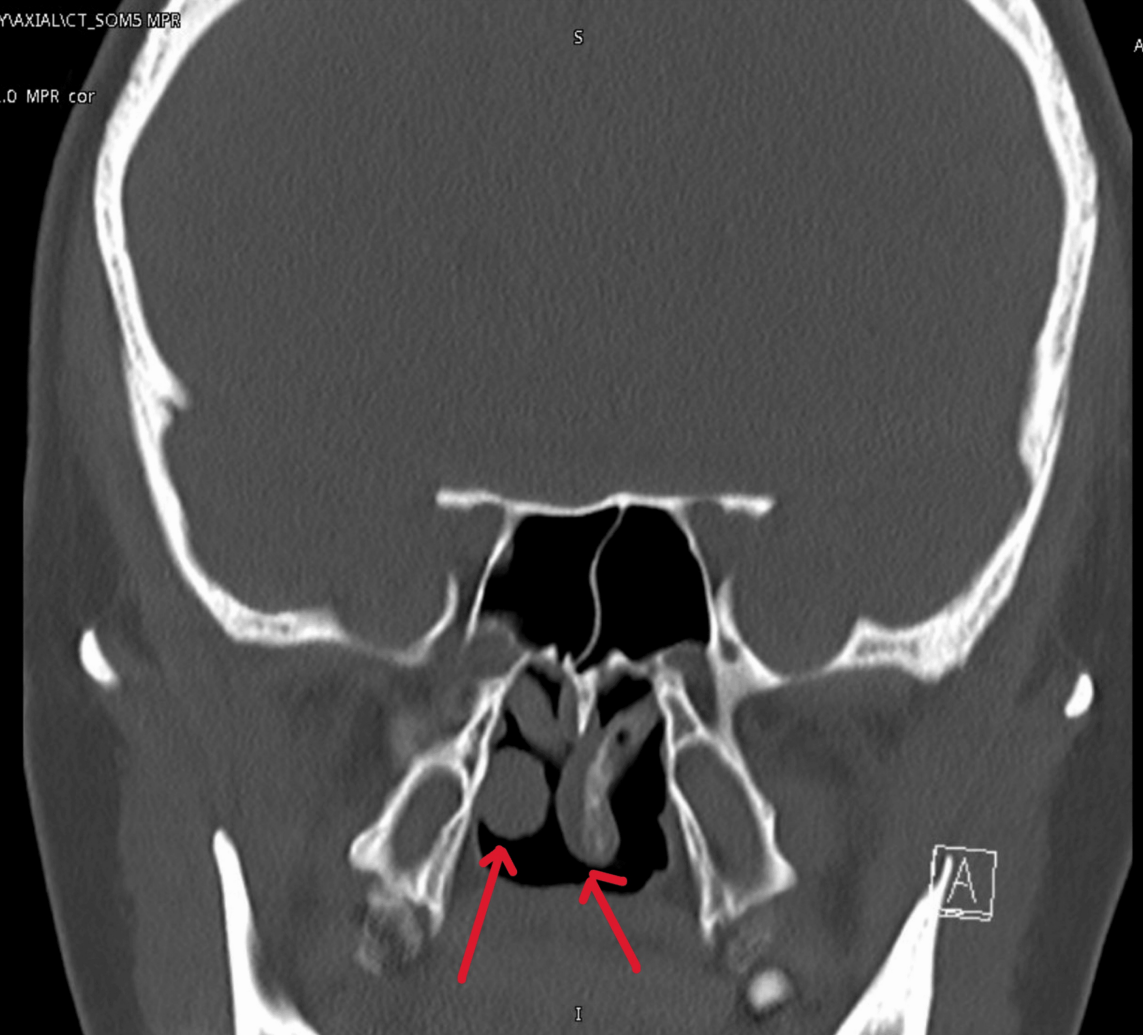
Postoperative CT scan with hanging middle turbinates in the nasopharynx

**Figure 4:**
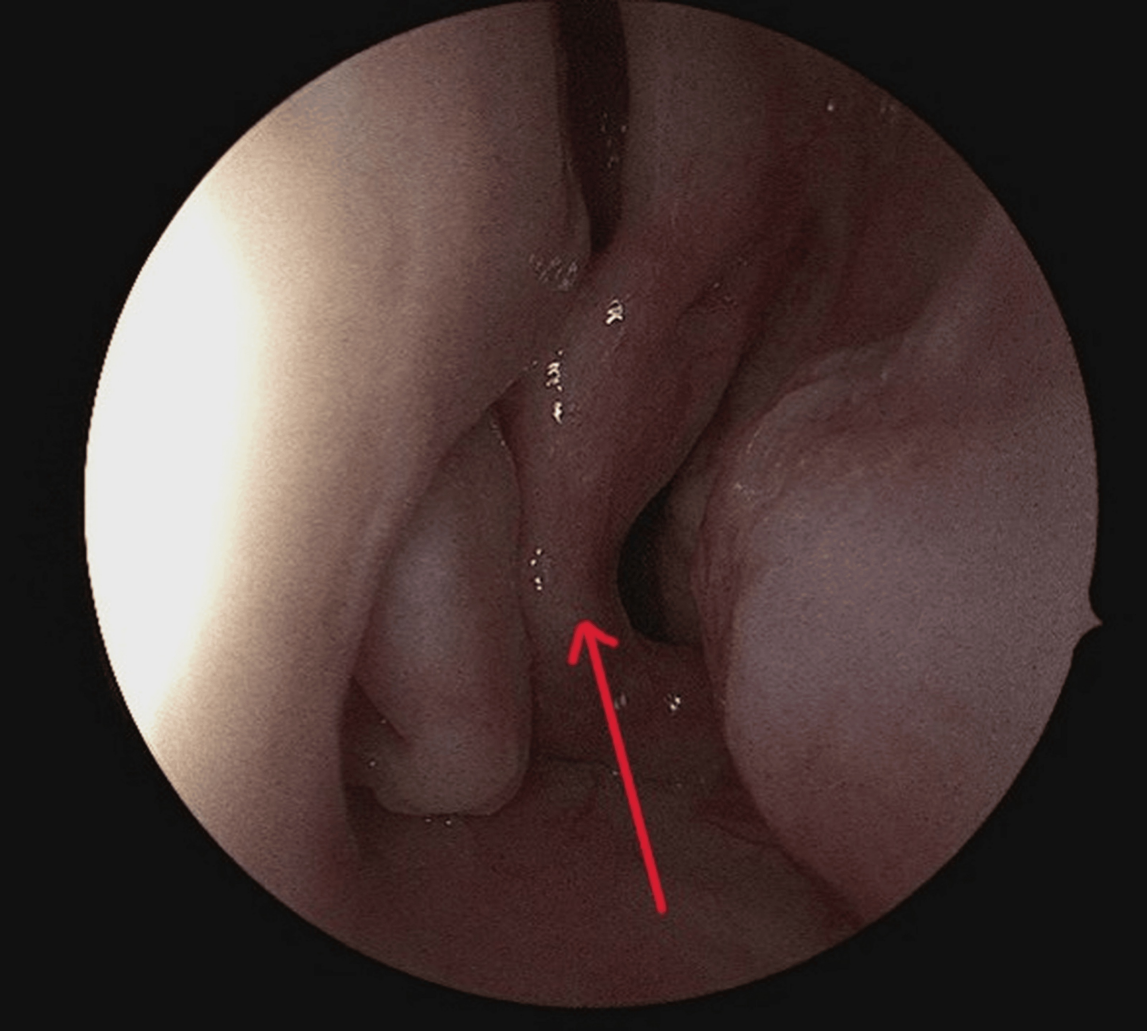
Intraoperative finding showing hanging middle turbinates in the nasopharynx (note the red arrow)

In addition, Figure 3 showed hanging middle turbinates into the nasopharynx. She was advised to undergo surgery, where she underwent revision septoplasty, excision of both hanging middle turbinates bilaterally with hemostasis and bilateral submucosal diathermy of the inferior turbinates. The intraoperative finding (Figure 4) showed bilateral hanging middle turbinates extending into the nasopharynx, along with a deviated nasal septum to the right. Notably, the lower part of the septal cartilage anteriorly is missing, and the maxillary crest is deviated to the right. The mucosa appears thin, with deviations involving both bone and cartilage posteriorly and superiorly. Additionally, there are enlarged inferior turbinates bilaterally. Postoperatively, the patient stayed one night at our facility and was then discharged the next day without any complications. Postoperatively, she had a smooth recovery with significant improvement in breathing and resolution of nasal obstruction.

## Discussion

This case highlights a rare otolaryngologic complication following septoplasty, presenting as persistent nasal obstruction caused by a hanging middle turbinate. In the literature, complications after septoplasty, such as infection in 3% of cases and bleeding in 6% of cases, pain and discomfort, ocular complications, septal abscess rates from 0.4% to 12.0%, and nasal septal perforation ranging from 1.6% to 6.7%, have been reported [2]. However, long-term complications, such as persistence of nasal airway obstruction requiring revision surgery, saddle nose deformity, anosmia, empty nose syndrome, CSF leakage, and even blindness and rarely oronasal fistula, are not well-studied in the literature [3,4]. There is no case report or literature that has discussed postoperative middle turbinates hanging in the nasopharynx as a complication of post septoplasty operation. This case highlights the importance of doing a preoperative CT scan in a revision septoplasty case, as this will help surgeons understand the anatomy that they are dealing with in surgery. This case enlightens otolaryngology surgeons to the presentations of complications that a patient can present after any septoplasty surgery. In addition, this case underscores the importance of having a radiological investigation before undergoing any revision corrective surgery. Not only will it help surgeons navigate through the anatomy, but also ensure that no other pathology is present. A limitation of this report is the absence of long-term follow-up data, which could offer valuable insight into the patient’s response and potential risk of relapse. This case emphasizes the importance of clinicians maintaining vigilance for atypical presentations of complications following septoplasty. We hypothesize that during surgery, turbinectomy scissors were mistakenly applied to the middle turbinate, which was initially thought to be the inferior turbinate. Upon recognition of the error, the procedure was stopped, resulting in a hanging middle turbinate. However, further research is needed to investigate the underlying causes of such postoperative complications. This case highlights the importance of anatomical knowledge, appropriate surgical training, and adherence to anatomical landmarks during turbinate surgery. These factors are particularly critical in revision septoplasty, where altered anatomy may increase the risk of uncommon but clinically significant complications.

## Conclusions

This case emphasizes the importance of clinicians maintaining vigilance for atypical presentations of complications following septoplasty. It also highlights the importance of doing a preoperative CT scan in revision septoplasty cases, as this will help surgeons understand the anatomy they are dealing with in surgery.
